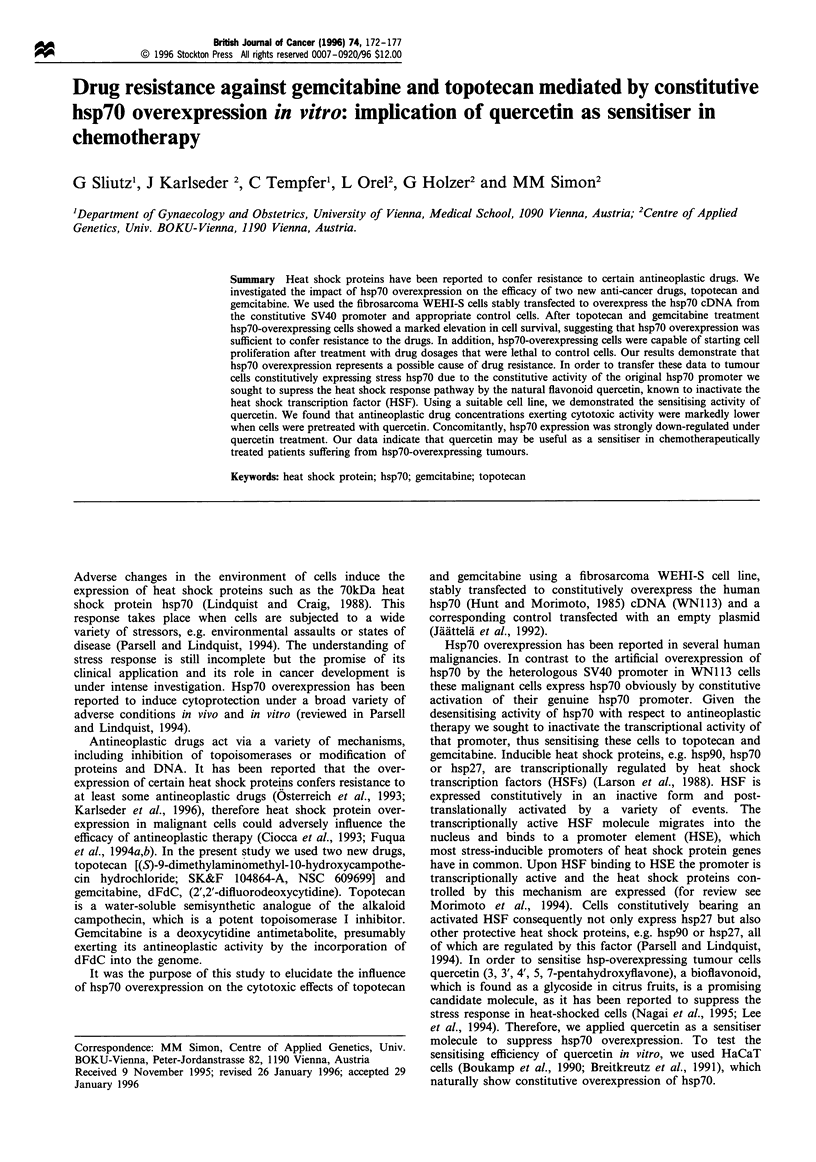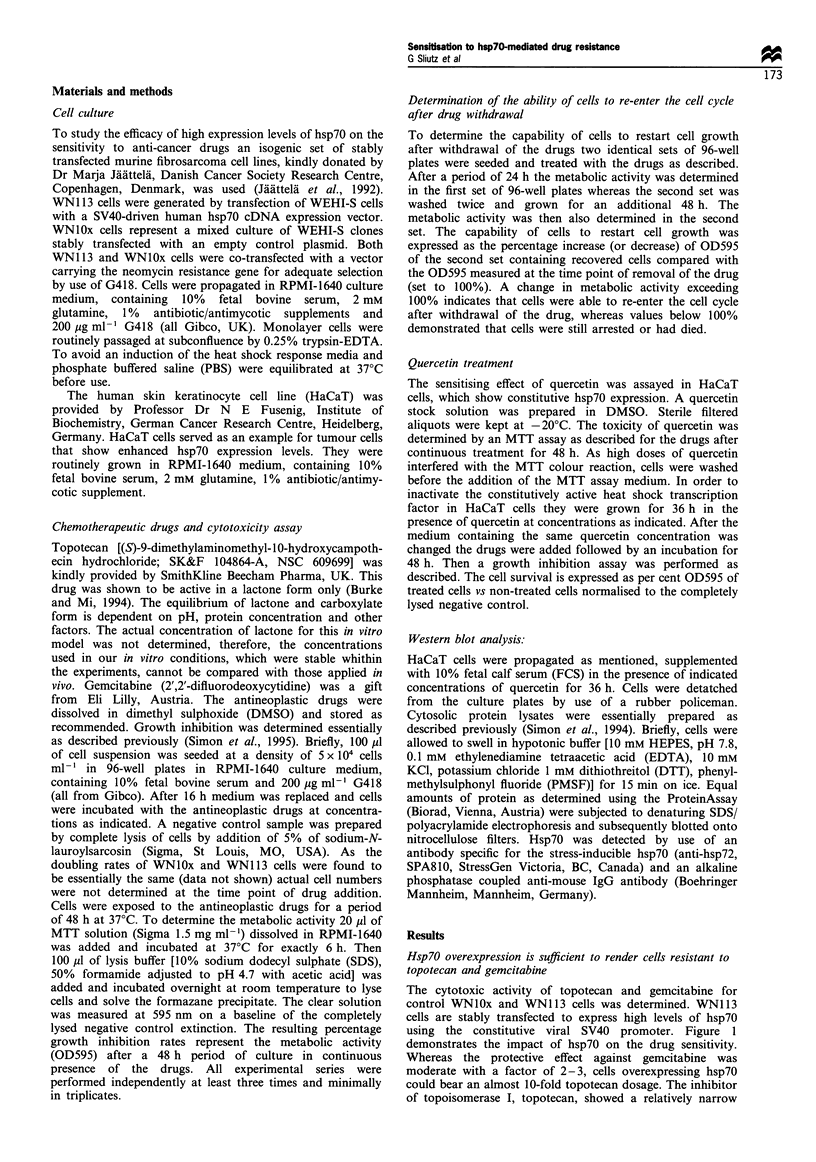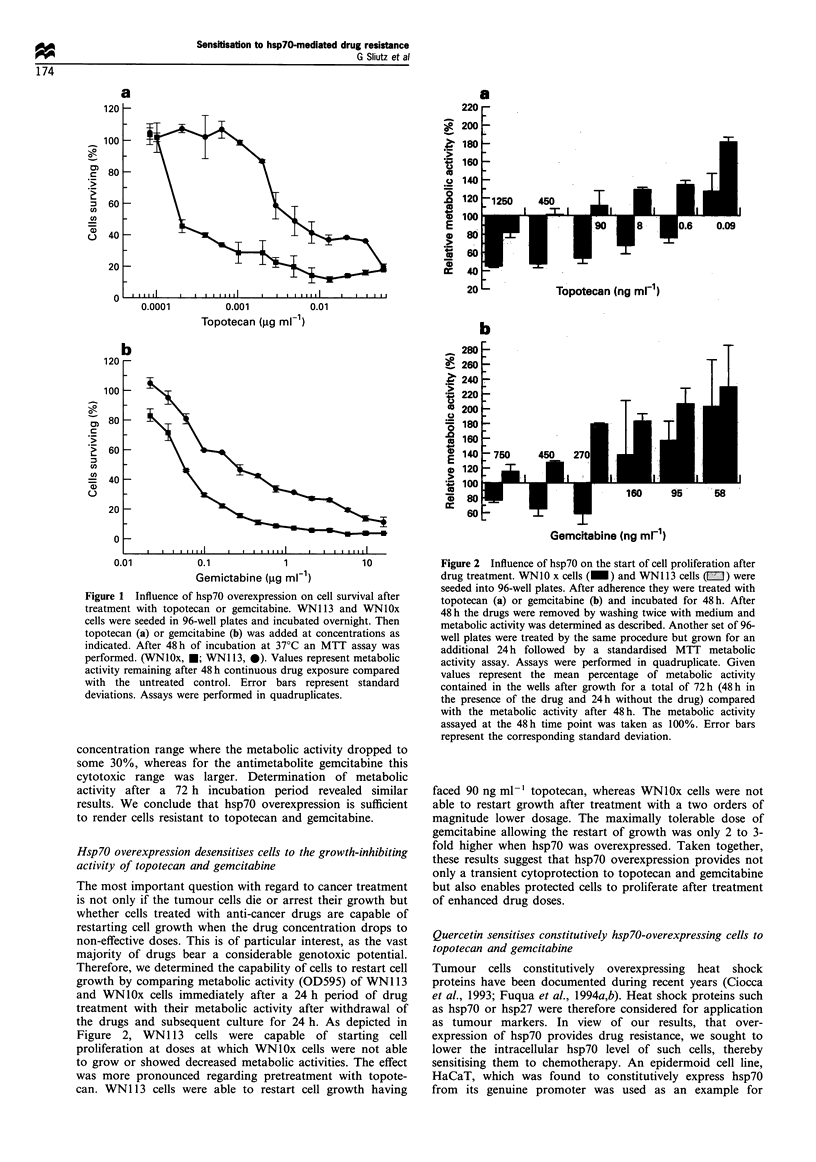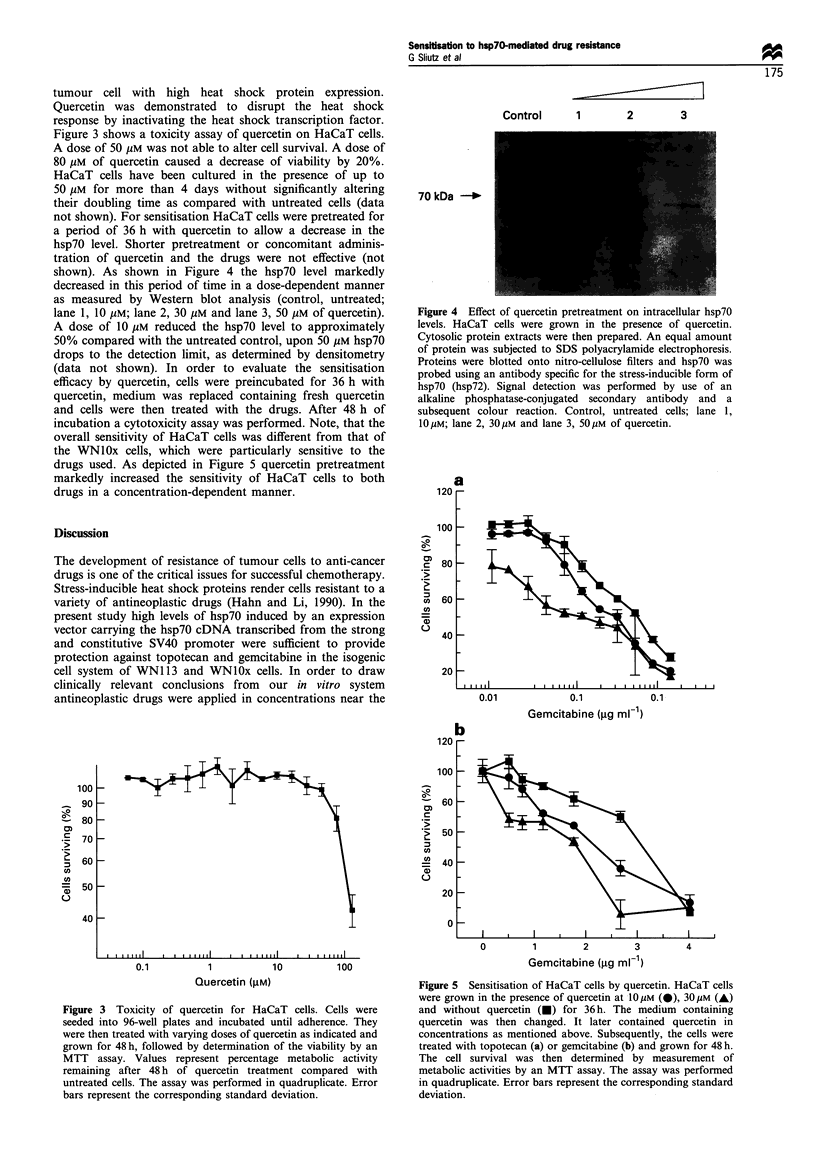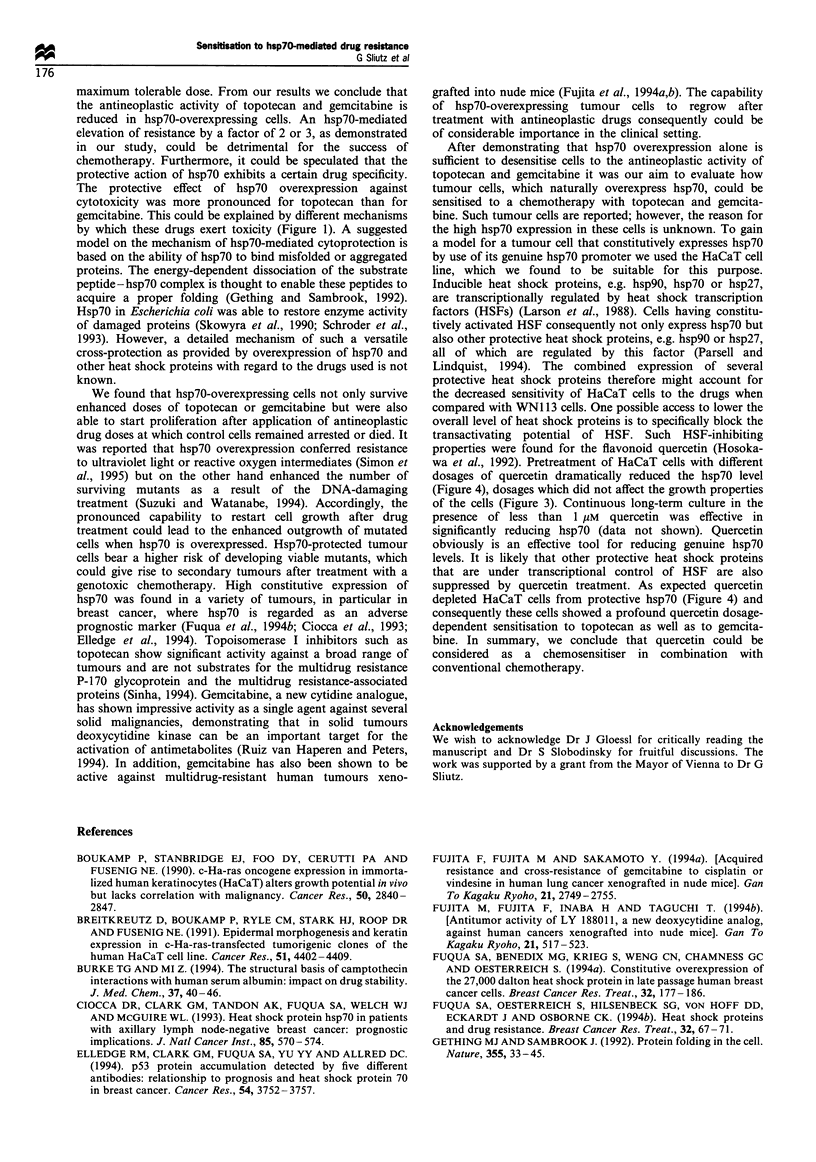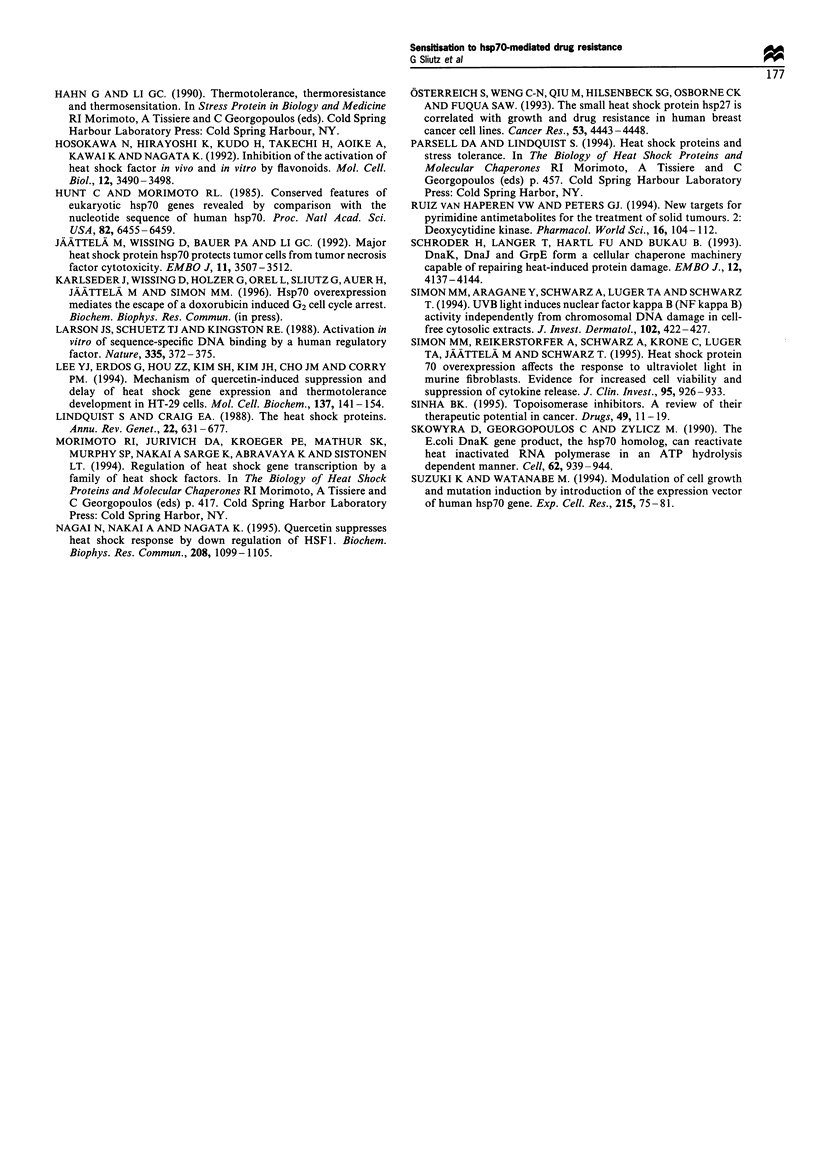# Drug resistance against gemcitabine and topotecan mediated by constitutive hsp70 overexpression in vitro: implication of quercetin as sensitiser in chemotherapy.

**DOI:** 10.1038/bjc.1996.334

**Published:** 1996-07

**Authors:** G. Sliutz, J. Karlseder, C. Tempfer, L. Orel, G. Holzer, M. M. Simon

**Affiliations:** Department of Gynaecology and Obstetrics, University of Vienna, Medical School, Austria.

## Abstract

**Images:**


British Journal of Cancer (1996) 74, 172-177
© 1996 Stockton Press All rights reserved 0007-0920/96 $12.00

### Drug resistance against gemcitabine and topotecan mediated by constitutive hsp70 overexpression *in vitro*: implication of quercetin as sensitiser in chemotherapy

G Sliutz[1], J Karlseder [2], C Tempfer[1], L Orel[2], G Holzer[2] and MM Simon[2]

[1]*Department of Gynaecology and Obstetrics, University of Vienna, Medical School, 1090 Vienna, Austria;* [2]*Centre of Applied Genetics, Univ. BOKU-Vienna, 1190 Vienna, Austria.*

**Summary** Heat shock proteins have been reported to confer resistance to certain antineoplastic drugs. We investigated the impact of hsp70 overexpression on the efficacy of two new anti-cancer drugs, topotecan and gemcitabine. We used the fibrosarcoma WEHI-S cells stably transfected to overexpress the hsp70 cDNA from the constitutive SV40 promoter and appropriate control cells. After topotecan and gemcitabine treatment hsp70-overexpressing cells showed a marked elevation in cell survival, suggesting that hsp70 overexpression was sufficient to confer resistance to the drugs. In addition, hsp70-overexpressing cells were capable of starting cell proliferation after treatment with drug dosages that were lethal to control cells. Our results demonstrate that hsp70 overexpression represents a possible cause of drug resistance. In order to transfer these data to tumour cells constitutively expressing stress hsp70 due to the constitutive activity of the original hsp70 promoter we sought to supress the heat shock response pathway by the natural flavonoid quercetin, known to inactivate the heat shock transcription factor (HSF). Using a suitable cell line, we demonstrated the sensitising activity of quercetin. We found that antineoplastic drug concentrations exerting cytotoxic activity were markedly lower when cells were pretreated with quercetin. Concomitantly, hsp70 expression was strongly down-regulated under quercetin treatment. Our data indicate that quercetin may be useful as a sensitiser in chemotherapeutically treated patients suffering from hsp70-overexpressing tumours.

**Keywords:** heat shock protein; hsp70; gemcitabine; topotecan

Adverse changes in the environment of cells induce the expression of heat shock proteins such as the 70kDa heat shock protein hsp70 (Lindquist and Craig, 1988). This response takes place when cells are subjected to a wide variety of stressors, e.g. environmental assaults or states of disease (Parsell and Lindquist, 1994). The understanding of stress response is still incomplete but the promise of its clinical application and its role in cancer development is under intense investigation. Hsp70 overexpression has been reported to induce cytoprotection under a broad variety of adverse conditions *in vivo* and *in vitro* (reviewed in Parsell and Lindquist, 1994).

Antineoplastic drugs act via a variety of mechanisms, including inhibition of topoisomerases or modification of proteins and DNA. It has been reported that the overexpression of certain heat shock proteins confers resistance to at least some antineoplastic drugs (Österreich *et al.*, 1993; Karlseder *et al.*, 1996), therefore heat shock protein overexpression in malignant cells could adversely influence the efficacy of antineoplastic therapy (Ciocca *et al.*, 1993; Fuqua *et al.*, 1994*a,b*). In the present study we used two new drugs, topotecan [(*S*)-9-dimethylaminomethyl-10-hydroxycampothecin hydrochloride; SK&F 104864-A, NSC 609699] and gemcitabine, dFdC, (2′,2′-difluorodeoxycytidine). Topotecan is a water-soluble semisynthetic analogue of the alkaloid campothecin, which is a potent topoisomerase I inhibitor. Gemcitabine is a deoxycytidine antimetabolite, presumably exerting its antineoplastic activity by the incorporation of dFdC into the genome.

It was the purpose of this study to elucidate the influence of hsp70 overexpression on the cytotoxic effects of topotecan and gemcitabine using a fibrosarcoma WEHI-S cell line, stably transfected to constitutively overexpress the human hsp70 (Hunt and Morimoto, 1985) cDNA (WN113) and a corresponding control transfected with an empty plasmid (Jäättelä *et al.*, 1992).

Hsp70 overexpression has been reported in several human malignancies. In contrast to the artificial overexpression of hsp70 by the heterologous SV40 promoter in WN113 cells these malignant cells express hsp70 obviously by constitutive activation of their genuine hsp70 promoter. Given the desensitising activity of hsp70 with respect to antineoplastic therapy we sought to inactivate the transcriptional activity of that promoter, thus sensitising these cells to topotecan and gemcitabine. Inducible heat shock proteins, e.g. hsp90, hsp70 or hsp27, are transcriptionally regulated by heat shock transcription factors (HSFs) (Larson *et al.*, 1988). HSF is expressed constitutively in an inactive form and post-translationally activated by a variety of events. The transcriptionally active HSF molecule migrates into the nucleus and binds to a promoter element (HSE), which most stress-inducible promoters of heat shock protein genes have in common. Upon HSF binding to HSE the promoter is transcriptionally active and the heat shock proteins controlled by this mechanism are expressed (for review see Morimoto *et al.*, 1994). Cells constitutively bearing an activated HSF consequently not only express hsp27 but also other protective heat shock proteins, e.g. hsp90 or hsp27, all of which are regulated by this factor (Parsell and Lindquist, 1994). In order to sensitise hsp-overexpressing tumour cells quercetin (3, 3′, 4′, 5, 7-pentahydroxyflavone), a bioflavonoid, which is found as a glycoside in citrus fruits, is a promising candidate molecule, as it has been reported to suppress the stress response in heat-shocked cells (Nagai *et al.*, 1995; Lee *et al.*, 1994). Therefore, we applied quercetin as a sensitiser molecule to suppress hsp70 overexpression. To test the sensitising efficiency of quercetin *in vitro*, we used HaCaT cells (Boukamp *et al.*, 1990; Breitkreutz *et al.*, 1991), which naturally show constitutive overexpression of hsp70.

Correspondence: MM Simon, Centre of Applied Genetics, Univ. BOKU-Vienna, Peter-Jordanstrasse 82, 1190 Vienna, Austria
Received 9 November 1995; revised 26 January 1996; accepted 29 January 1996

### Materials and methods

### *Cell culture*

To study the efficacy of high expression levels of hsp70 on the sensitivity to anti-cancer drugs an isogenic set of stably transfected murine fibrosarcoma cell lines, kindly donated by Dr Marja Jäättelä, Danish Cancer Society Research Centre, Copenhagen, Denmark, was used (Jäättelä *et al.*, 1992). WN113 cells were generated by transfection of WEHI-S cells with a SV40-driven human hsp70 cDNA expression vector. WN10x cells represent a mixed culture of WEHI-S clones stably transfected with an empty control plasmid. Both WN113 and WN10x cells were co-transfected with a vector carrying the neomycin resistance gene for adequate selection by use of G418. Cells were propagated in RPMI-1640 culture medium, containing 10% fetal bovine serum, 2 mM glutamine, 1% antibiotic/antimycotic supplements and 200 $\mu g\ ml^{-1}$ G418 (all Gibco, UK). Monolayer cells were routinely passaged at subconfluence by 0.25% trypsin-EDTA. To avoid an induction of the heat shock response media and phosphate buffered saline (PBS) were equilibrated at 37°C before use.

The human skin keratinocyte cell line (HaCaT) was provided by Professor Dr N E Fusenig, Institute of Biochemistry, German Cancer Research Centre, Heidelberg, Germany. HaCaT cells served as an example for tumour cells that show enhanced hsp70 expression levels. They were routinely grown in RPMI-1640 medium, containing 10% fetal bovine serum, 2 mM glutamine, 1% antibiotic/antimycotic supplement.

### *Chemotherapeutic drugs and cytotoxicity assay*

Topotecan [(*S*)-9-dimethylaminomethyl-10-hydroxycampothecin hydrochloride; SK&F 104864-A, NSC 609699] was kindly provided by SmithKline Beecham Pharma, UK. This drug was shown to be active in a lactone form only (Burke and Mi, 1994). The equilibrium of lactone and carboxylate form is dependent on pH, protein concentration and other factors. The actual concentration of lactone for this *in vitro* model was not determined, therefore, the concentrations used in our *in vitro* conditions, which were stable whithin the experiments, cannot be compared with those applied *in vivo*. Gemcitabine (2′,2′-difluorodeoxycytidine) was a gift from Eli Lilly, Austria. The antineoplastic drugs were dissolved in dimethyl sulphoxide (DMSO) and stored as recommended. Growth inhibition was determined essentially as described previously (Simon *et al.*, 1995). Briefly, 100 $\mu l$ of cell suspension was seeded at a density of $5\times10^4$ cells $ml^{-1}$ in 96-well plates in RPMI-1640 culture medium, containing 10% fetal bovine serum and 200 $\mu g\ ml^{-1}$ G418 (all from Gibco). After 16 h medium was replaced and cells were incubated with the antineoplastic drugs at concentrations as indicated. A negative control sample was prepared by complete lysis of cells by addition of 5% of sodium-*N*-lauroylsarcosin (Sigma, St Louis, MO, USA). As the doubling rates of WN10x and WN113 cells were found to be essentially the same (data not shown) actual cell numbers were not determined at the time point of drug addition. Cells were exposed to the antineoplastic drugs for a period of 48 h at 37°C. To determine the metabolic activity 20 $\mu l$ of MTT solution (Sigma 1.5 mg $ml^{-1}$) dissolved in RPMI-1640 was added and incubated at 37°C for exactly 6 h. Then 100 $\mu l$ of lysis buffer [10% sodium dodecyl sulphate (SDS), 50% formamide adjusted to pH 4.7 with acetic acid] was added and incubated overnight at room temperature to lyse cells and solve the formazane precipitate. The clear solution was measured at 595 nm on a baseline of the completely lysed negative control extinction. The resulting percentage growth inhibition rates represent the metabolic activity (OD595) after a 48 h period of culture in continuous presence of the drugs. All experimental series were performed independently at least three times and minimally in triplicates.

### *Determination of the ability of cells to re-enter the cell cycle after drug withdrawal*

To determine the capability of cells to restart cell growth after withdrawal of the drugs two identical sets of 96-well plates were seeded and treated with the drugs as described. After a period of 24 h the metabolic activity was determined in the first set of 96-well plates whereas the second set was washed twice and grown for an additional 48 h. The metabolic activity was then also determined in the second set. The capability of cells to restart cell growth was expressed as the percentage increase (or decrease) of OD595 of the second set containing recovered cells compared with the OD595 measured at the time point of removal of the drug (set to 100%). A change in metabolic activity exceeding 100% indicates that cells were able to re-enter the cell cycle after withdrawal of the drug, whereas values below 100% demonstrated that cells were still arrested or had died.

### *Quercetin treatment*

The sensitising effect of quercetin was assayed in HaCaT cells, which show constitutive hsp70 expression. A quercetin stock solution was prepared in DMSO. Sterile filtered aliquots were kept at −20°C. The toxicity of quercetin was determined by an MTT assay as described for the drugs after continuous treatment for 48 h. As high doses of quercetin interfered with the MTT colour reaction, cells were washed before the addition of the MTT assay medium. In order to inactivate the constitutively active heat shock transcription factor in HaCaT cells they were grown for 36 h in the presence of quercetin at concentrations as indicated. After the medium containing the same quercetin concentration was changed the drugs were added followed by an incubation for 48 h. Then a growth inhibition assay was performed as described. The cell survival is expressed as per cent OD595 of treated cells *vs* non-treated cells normalised to the completely lysed negative control.

### *Western blot analysis:*

HaCaT cells were propagated as mentioned, supplemented with 10% fetal calf serum (FCS) in the presence of indicated concentrations of quercetin for 36 h. Cells were detatched from the culture plates by use of a rubber policeman. Cytosolic protein lysates were essentially prepared as described previously (Simon *et al.*, 1994). Briefly, cells were allowed to swell in hypotonic buffer [10 mM HEPES, pH 7.8, 0.1 mM ethylenediamine tetraacetic acid (EDTA), 10 mM KCl, potassium chloride 1 mM dithiothreitol (DTT), phenylmethylsulphonyl fluoride (PMSF)] for 15 min on ice. Equal amounts of protein as determined using the ProteinAssay (Biorad, Vienna, Austria) were subjected to denaturing SDS/polyacrylamide electrophoresis and subsequently blotted onto nitrocellulose filters. Hsp70 was detected by use of an antibody specific for the stress-inducible hsp70 (anti-hsp72, SPA810, StressGen Victoria, BC, Canada) and an alkaline phosphatase coupled anti-mouse IgG antibody (Boehringer Mannheim, Mannheim, Germany).

### Results

### *Hsp70 overexpression is sufficient to render cells resistant to topotecan and gemcitabine*

The cytotoxic activity of topotecan and gemcitabine for control WN10x and WN113 cells was determined. WN113 cells are stably transfected to express high levels of hsp70 using the constitutive viral SV40 promoter. Figure 1 demonstrates the impact of hsp70 on the drug sensitivity. Whereas the protective effect against gemcitabine was moderate with a factor of 2−3, cells overexpressing hsp70 could bear an almost 10-fold topotecan dosage. The inhibitor of topoisomerase I, topotecan, showed a relatively narrow

**Figure 1** Influence of hsp70 overexpression on cell survival after treatment with topotecan or gemcitabine. WN113 and WN10x cells were seeded in 96-well plates and incubated overnight. Then topotecan (**a**) or gemcitabine (**b**) was added at concentrations as indicated. After 48 h of incubation at 37°C an MTT assay was performed. (WN10x, ■; WN113, ●). Values represent metabolic activity remaining after 48 h continuous drug exposure compared with the untreated control. Error bars represent standard deviations. Assays were performed in quadruplicates.

**Figure 2** Influence of hsp70 on the start of cell proliferation after drug treatment. WN10 x cells (■) and WN113 cells (▒) were seeded into 96-well plates. After adherence they were treated with topotecan (**a**) or gemcitabine (**b**) and incubated for 48 h. After 48 h the drugs were removed by washing twice with medium and metabolic activity was determined as described. Another set of 96-well plates were treated by the same procedure but grown for an additional 24 h followed by a standardised MTT metabolic activity assay. Assays were performed in quadruplicate. Given values represent the mean percentage of metabolic activity contained in the wells after growth for a total of 72 h (48 h in the presence of the drug and 24 h without the drug) compared with the metabolic activity after 48 h. The metabolic activity assayed at the 48 h time point was taken as 100%. Error bars represent the corresponding standard deviation.

concentration range where the metabolic activity dropped to some 30%, whereas for the antimetabolite gemcitabine this cytotoxic range was larger. Determination of metabolic activity after a 72 h incubation period revealed similar results. We conclude that hsp70 overexpression is sufficient to render cells resistant to topotecan and gemcitabine.

### *Hsp70 overexpression desensitises cells to the growth-inhibiting activity of topotecan and gemcitabine*

The most important question with regard to cancer treatment is not only if the tumour cells die or arrest their growth but whether cells treated with anti-cancer drugs are capable of restarting cell growth when the drug concentration drops to non-effective doses. This is of particular interest, as the vast majority of drugs bear a considerable genotoxic potential. Therefore, we determined the capability of cells to restart cell growth by comparing metabolic activity (OD595) of WN113 and WN10x cells immediately after a 24 h period of drug treatment with their metabolic activity after withdrawal of the drugs and subsequent culture for 24 h. As depicted in Figure 2, WN113 cells were capable of starting cell proliferation at doses at which WN10x cells were not able to grow or showed decreased metabolic activities. The effect was more pronounced regarding pretreatment with topotecan. WN113 cells were able to restart cell growth having faced 90 ng ml$^{-1}$ topotecan, whereas WN10x cells were not able to restart growth after treatment with a two orders of magnitude lower dosage. The maximally tolerable dose of gemcitabine allowing the restart of growth was only 2 to 3-fold higher when hsp70 was overexpressed. Taken together, these results suggest that hsp70 overexpression provides not only a transient cytoprotection to topotecan and gemcitabine but also enables protected cells to proliferate after treatment of enhanced drug doses.

### *Quercetin sensitises constitutively hsp70-overexpressing cells to topotecan and gemcitabine*

Tumour cells constitutively overexpressing heat shock proteins have been documented during recent years (Ciocca *et al.*, 1993; Fuqua *et al.*, 1994*a*,*b*). Heat shock proteins such as hsp70 or hsp27 were therefore considered for application as tumour markers. In view of our results, that overexpression of hsp70 provides drug resistance, we sought to lower the intracellular hsp70 level of such cells, thereby sensitising them to chemotherapy. An epidermoid cell line, HaCaT, which was found to constitutively express hsp70 from its genuine promoter was used as an example for tumour cell with high heat shock protein expression. Quercetin was demonstrated to disrupt the heat shock response by inactivating the heat shock transcription factor. Figure 3 shows a toxicity assay of quercetin on HaCaT cells. A dose of 50 μM was not able to alter cell survival. A dose of 80 μM of quercetin caused a decrease of viability by 20%. HaCaT cells have been cultured in the presence of up to 50 μM for more than 4 days without significantly altering their doubling time as compared with untreated cells (data not shown). For sensitisation HaCaT cells were pretreated for a period of 36 h with quercetin to allow a decrease in the hsp70 level. Shorter pretreatment or concomitant administration of quercetin and the drugs were not effective (not shown). As shown in Figure 4 the hsp70 level markedly decreased in this period of time in a dose-dependent manner as measured by Western blot analysis (control, untreated; lane 1, 10 μM; lane 2, 30 μM and lane 3, 50 μM of quercetin). A dose of 10 μM reduced the hsp70 level to approximately 50% compared with the untreated control, upon 50 μM hsp70 drops to the detection limit, as determined by densitometry (data not shown). In order to evaluate the sensitisation efficacy by quercetin, cells were preincubated for 36 h with quercetin, medium was replaced containing fresh quercetin and cells were then treated with the drugs. After 48 h of incubation a cytotoxicity assay was performed. Note, that the overall sensitivity of HaCaT cells was different from that of the WN10x cells, which were particularly sensitive to the drugs used. As depicted in Figure 5 quercetin pretreatment markedly increased the sensitivity of HaCaT cells to both drugs in a concentration-dependent manner.

### Discussion

The development of resistance of tumour cells to anti-cancer drugs is one of the critical issues for successful chemotherapy. Stress-inducible heat shock proteins render cells resistant to a variety of antineoplastic drugs (Hahn and Li, 1990). In the present study high levels of hsp70 induced by an expression vector carrying the hsp70 cDNA transcribed from the strong and constitutive SV40 promoter were sufficient to provide protection against topotecan and gemcitabine in the isogenic cell system of WN113 and WN10x cells. In order to draw clinically relevant conclusions from our *in vitro* system antineoplastic drugs were applied in concentrations near the

**Figure 3** Toxicity of quercetin for HaCaT cells. Cells were seeded into 96-well plates and incubated until adherence. They were then treated with varying doses of quercetin as indicated and grown for 48 h, followed by determination of the viability by an MTT assay. Values represent percentage metabolic activity remaining after 48 h of quercetin treatment compared with untreated cells. The assay was performed in quadruplicate. Error bars represent the corresponding standard deviation.

**Figure 4** Effect of quercetin pretreatment on intracellular hsp70 levels. HaCaT cells were grown in the presence of quercetin. Cytosolic protein extracts were then prepared. An equal amount of protein was subjected to SDS polyacrylamide electrophoresis. Proteins were blotted onto nitro-cellulose filters and hsp70 was probed using an antibody specific for the stress-inducible form of hsp70 (hsp72). Signal detection was performed by use of an alkaline phosphatase-conjugated secondary antibody and a subsequent colour reaction. Control, untreated cells; lane 1, 10 μM; lane 2, 30 μM and lane 3, 50 μM of quercetin.

**Figure 5** Sensitisation of HaCaT cells by quercetin. HaCaT cells were grown in the presence of quercetin at 10 μM (●), 30 μM (▲) and without quercetin (■) for 36 h. The medium containing quercetin was then changed. It later contained quercetin in concentrations as mentioned above. Subsequently, the cells were treated with topotecan (**a**) or gemcitabine (**b**) and grown for 48 h. The cell survival was then determined by measurement of metabolic activities by an MTT assay. The assay was performed in quadruplicate. Error bars represent the corresponding standard deviation.

maximum tolerable dose. From our results we conclude that the antineoplastic activity of topotecan and gemcitabine is reduced in hsp70-overexpressing cells. An hsp70-mediated elevation of resistance by a factor of 2 or 3, as demonstrated in our study, could be detrimental for the success of chemotherapy. Furthermore, it could be speculated that the protective action of hsp70 exhibits a certain drug specificity. The protective effect of hsp70 overexpression against cytotoxicity was more pronounced for topotecan than for gemcitabine. This could be explained by different mechanisms by which these drugs exert toxicity (Figure 1). A suggested model on the mechanism of hsp70-mediated cytoprotection is based on the ability of hsp70 to bind misfolded or aggregated proteins. The energy-dependent dissociation of the substrate peptide–hsp70 complex is thought to enable these peptides to acquire a proper folding (Gething and Sambrook, 1992). Hsp70 in *Escherichia coli* was able to restore enzyme activity of damaged proteins (Skowyra *et al.*, 1990; Schroder *et al.*, 1993). However, a detailed mechanism of such a versatile cross-protection as provided by overexpression of hsp70 and other heat shock proteins with regard to the drugs used is not known.

We found that hsp70-overexpressing cells not only survive enhanced doses of topotecan or gemcitabine but were also able to start proliferation after application of antineoplastic drug doses at which control cells remained arrested or died. It was reported that hsp70 overexpression conferred resistance to ultraviolet light or reactive oxygen intermediates (Simon *et al.*, 1995) but on the other hand enhanced the number of surviving mutants as a result of the DNA-damaging treatment (Suzuki and Watanabe, 1994). Accordingly, the pronounced capability to restart cell growth after drug treatment could lead to the enhanced outgrowth of mutated cells when hsp70 is overexpressed. Hsp70-protected tumour cells bear a higher risk of developing viable mutants, which could give rise to secondary tumours after treatment with a genotoxic chemotherapy. High constitutive expression of hsp70 was found in a variety of tumours, in particular in breast cancer, where hsp70 is regarded as an adverse prognostic marker (Fuqua *et al.*, 1994*b*; Ciocca *et al.*, 1993; Elledge *et al.*, 1994). Topoisomerase I inhibitors such as topotecan show significant activity against a broad range of tumours and are not substrates for the multidrug resistance P-170 glycoprotein and the multidrug resistance-associated proteins (Sinha, 1994). Gemcitabine, a new cytidine analogue, has shown impressive activity as a single agent against several solid malignancies, demonstrating that in solid tumours deoxycytidine kinase can be an important target for the activation of antimetabolites (Ruiz van Haperen and Peters, 1994). In addition, gemcitabine has also been shown to be active against multidrug-resistant human tumours xenografted into nude mice (Fujita *et al.*, 1994*a,b*). The capability of hsp70-overexpressing tumour cells to regrow after treatment with antineoplastic drugs consequently could be of considerable importance in the clinical setting.

After demonstrating that hsp70 overexpression alone is sufficient to desensitise cells to the antineoplastic activity of topotecan and gemcitabine it was our aim to evaluate how tumour cells, which naturally overexpress hsp70, could be sensitised to a chemotherapy with topotecan and gemcitabine. Such tumour cells are reported; however, the reason for the high hsp70 expression in these cells is unknown. To gain a model for a tumour cell that constitutively expresses hsp70 by use of its genuine hsp70 promoter we used the HaCaT cell line, which we found to be suitable for this purpose. Inducible heat shock proteins, e.g. hsp90, hsp70 or hsp27, are transcriptionally regulated by heat shock transcription factors (HSFs) (Larson *et al.*, 1988). Cells having constitutively activated HSF consequently not only express hsp70 but also other protective heat shock proteins, e.g. hsp90 or hsp27, all of which are regulated by this factor (Parsell and Lindquist, 1994). The combined expression of several protective heat shock proteins therefore might account for the decreased sensitivity of HaCaT cells to the drugs when compared with WN113 cells. One possible access to lower the overall level of heat shock proteins is to specifically block the transactivating potential of HSF. Such HSF-inhibiting properties were found for the flavonoid quercetin (Hosokawa *et al.*, 1992). Pretreatment of HaCaT cells with different dosages of quercetin dramatically reduced the hsp70 level (Figure 4), dosages which did not affect the growth properties of the cells (Figure 3). Continuous long-term culture in the presence of less than 1 $\mu$M quercetin was effective in significantly reducing hsp70 (data not shown). Quercetin obviously is an effective tool for reducing genuine hsp70 levels. It is likely that other protective heat shock proteins that are under transcriptional control of HSF are also suppressed by quercetin treatment. As expected quercetin depleted HaCaT cells from protective hsp70 (Figure 4) and consequently these cells showed a profound quercetin dosage-dependent sensitisation to topotecan as well as to gemcitabine. In summary, we conclude that quercetin could be considered as a chemosensitiser in combination with conventional chemotherapy.

**Acknowledgements**
We wish to acknowledge Dr J Gloessl for critically reading the manuscript and Dr S Slobodinsky for fruitful discussions. The work was supported by a grant from the Mayor of Vienna to Dr G Sliutz.

### References

BOUKAMP P, STANBRIDGE EJ, FOO DY, CERUTTI PA AND FUSENIG NE. (1990). c-Ha-ras oncogene expression in immortalized human keratinocytes (HaCaT) alters growth potential *in vivo* but lacks correlation with malignancy. *Cancer Res.*, **50**, 2840–2847.

BREITKREUTZ D, BOUKAMP P, RYLE CM, STARK HJ, ROOP DR AND FUSENIG NE. (1991). Epidermal morphogenesis and keratin expression in c-Ha-ras-transfected tumorigenic clones of the human HaCaT cell line. *Cancer Res.*, **51**, 4402–4409.

BURKE TG AND MI Z. (1994). The structural basis of camptothecin interactions with human serum albumin: impact on drug stability. *J. Med. Chem.*, **37**, 40–46.

CIOCCA DR, CLARK GM, TANDON AK, FUQUA SA, WELCH WJ AND MCGUIRE WL. (1993). Heat shock protein hsp70 in patients with axillary lymph node-negative breast cancer: prognostic implications. *J. Natl Cancer Inst.*, **85**, 570–574.

ELLEDGE RM, CLARK GM, FUQUA SA, YU YY AND ALLRED DC. (1994). p53 protein accumulation detected by five different antibodies: relationship to prognosis and heat shock protein 70 in breast cancer. *Cancer Res.*, **54**, 3752–3757.

FUJITA F, FUJITA M AND SAKAMOTO Y. (1994*a*). [Acquired resistance and cross-resistance of gemcitabine to cisplatin or vindesine in human lung cancer xenografted in nude mice]. *Gan To Kagaku Ryoho*, **21**, 2749–2755.

FUJITA M, FUJITA F, INABA H AND TAGUCHI T. (1994*b*). [Antitumor activity of LY 188011, a new deoxycytidine analog, against human cancers xenografted into nude mice]. *Gan To Kagaku Ryoho*, **21**, 517–523.

FUQUA SA, BENEDIX MG, KRIEG S, WENG CN, CHAMNESS GC AND OESTERREICH S. (1994*a*). Constitutive overexpression of the 27,000 dalton heat shock protein in late passage human breast cancer cells. *Breast Cancer Res. Treat.*, **32**, 177–186.

FUQUA SA, OESTERREICH S, HILSENBECK SG, VON HOFF DD, ECKARDT J AND OSBORNE CK. (1994*b*). Heat shock proteins and drug resistance. *Breast Cancer Res. Treat.*, **32**, 67–71.

GETHING MJ AND SAMBROOK J. (1992). Protein folding in the cell. *Nature*, **355**, 33–45.

HAHN G AND LI GC. (1990). Thermotolerance, thermoresistance and thermosensitation. In *Stress Protein in Biology and Medicine* RI Morimoto, A Tissiere and C Georgopoulos (eds). Cold Spring Harbour Laboratory Press: Cold Spring Harbour, NY.

HOSOKAWA N, HIRAYOSHI K, KUDO H, TAKECHI H, AOIKE A, KAWAI K AND NAGATA K. (1992). Inhibition of the activation of heat shock factor *in vivo* and *in vitro* by flavonoids. *Mol. Cell. Biol.*, **12,** 3490–3498.

HUNT C AND MORIMOTO RL. (1985). Conserved features of eukaryotic hsp70 genes revealed by comparison with the nucleotide sequence of human hsp70. *Proc. Natl Acad. Sci. USA*, **82,** 6455–6459.

JÄÄTTELÄ M, WISSING D, BAUER PA AND LI GC. (1992). Major heat shock protein hsp70 protects tumor cells from tumor necrosis factor cytotoxicity. *EMBO J*, **11,** 3507–3512.

KARLSEDER J, WISSING D, HOLZER G, OREL L, SLIUTZ G, AUER H, JÄÄTTELÄ M AND SIMON MM. (1996). Hsp70 overexpression mediates the escape of a doxorubicin induced $G_2$ cell cycle arrest. *Biochem. Biophys. Res. Commun.* (in press).

LARSON JS, SCHUETZ TJ AND KINGSTON RE. (1988). Activation *in vitro* of sequence-specific DNA binding by a human regulatory factor. *Nature*, **335,** 372–375.

LEE YJ, ERDOS G, HOU ZZ, KIM SH, KIM JH, CHO JM AND CORRY PM. (1994). Mechanism of quercetin-induced suppression and delay of heat shock gene expression and thermotolerance development in HT-29 cells. *Mol. Cell. Biochem.*, **137,** 141–154.

LINDQUIST S AND CRAIG EA. (1988). The heat shock proteins. *Annu. Rev. Genet.*, **22,** 631–677.

MORIMOTO RI, JURIVICH DA, KROEGER PE, MATHUR SK, MURPHY SP, NAKAI A SARGE K, ABRAVAYA K AND SISTONEN LT. (1994). Regulation of heat shock gene transcription by a family of heat shock factors. In *The Biology of Heat Shock Proteins and Molecular Chaperones* RI Morimoto, A Tissiere and C Georgopoulos (eds) p. 417. Cold Spring Harbor Laboratory Press: Cold Spring Harbor, NY.

NAGAI N, NAKAI A AND NAGATA K. (1995). Quercetin suppresses heat shock response by down regulation of HSF1. *Biochem. Biophys. Res. Commun.*, **208,** 1099–1105.

ÖSTERREICH S, WENG C-N, QIU M, HILSENBECK SG, OSBORNE CK AND FUQUA SAW. (1993). The small heat shock protein hsp27 is correlated with growth and drug resistance in human breast cancer cell lines. *Cancer Res.*, **53,** 4443–4448.

PARSELL DA AND LINDQUIST S. (1994). Heat shock proteins and stress tolerance. In *The Biology of Heat Shock Proteins and Molecular Chaperones* RI Morimoto, A Tissiere and C Georgopoulos (eds) p. 457. Cold Spring Harbour Laboratory Press: Cold Spring Harbor, NY.

RUIZ VAN HAPEREN VW AND PETERS GJ. (1994). New targets for pyrimidine antimetabolites for the treatment of solid tumours. 2: Deoxycytidine kinase. *Pharmacol. World Sci.*, **16,** 104–112.

SCHRODER H, LANGER T, HARTL FU AND BUKAU B. (1993). DnaK, DnaJ and GrpE form a cellular chaperone machinery capable of repairing heat-induced protein damage. *EMBO J.*, **12,** 4137–4144.

SIMON MM, ARAGANE Y, SCHWARZ A, LUGER TA AND SCHWARZ T. (1994). UVB light induces nuclear factor kappa B (NF kappa B) activity independently from chromosomal DNA damage in cell-free cytosolic extracts. *J. Invest. Dermatol.*, **102,** 422–427.

SIMON MM, REIKERSTORFER A, SCHWARZ A, KRONE C, LUGER TA, JÄÄTTELÄ M AND SCHWARZ T. (1995). Heat shock protein 70 overexpression affects the response to ultraviolet light in murine fibroblasts. Evidence for increased cell viability and suppression of cytokine release. *J. Clin. Invest.*, **95,** 926–933.

SINHA BK. (1995). Topoisomerase inhibitors. A review of their therapeutic potential in cancer. *Drugs*, **49,** 11–19.

SKOWYRA D, GEORGOPOULOS C AND ZYLICZ M. (1990). The E.coli DnaK gene product, the hsp70 homolog, can reactivate heat inactivated RNA polymerase in an ATP hydrolysis dependent manner. *Cell*, **62,** 939–944.

SUZUKI K AND WATANABE M. (1994). Modulation of cell growth and mutation induction by introduction of the expression vector of human hsp70 gene. *Exp. Cell. Res.*, **215,** 75–81.